# Assessing the effectiveness of rapamycin on angiomyolipoma in tuberous sclerosis: a two years trial

**DOI:** 10.1186/1750-1172-7-87

**Published:** 2012-11-11

**Authors:** Cristina Cabrera-López, Teresa Martí, Violeta Catalá, Ferran Torres, Silvia Mateu, Jose Ballarín, Roser Torra

**Affiliations:** 1 Department of Nephrology, Inherited Renal Diseases, Fundación Puigvert, Universidad Autónoma de Barcelona, Cartagena 340-350, Barcelona, 08025, Spain; 2 Department of Radiology, Fundación Puigvert, Universidad Autónoma de Barcelona, Cartagena 340-350, Barcelona, 08025, Spain; 3 Statistics and Methodological Support (USEM), IDIBAPS, Hospital Clínic, Mallorca 183, 08036, Barcelona, Spain; 4 Biostatistics Unit, Universidad Autónoma de Barcelona, Barcelona, Spain; 5 Coordination in Biomedical Research, Fundación Puigvert, Universidad Autónoma de Barcelona, Cartagena 340-350, Barcelona, 08025, Spain

**Keywords:** Angiomyolipoma, mTOR, Rapamycin, Treatment, Tuberous sclerosis

## Abstract

**Background:**

Tuberous sclerosis (TS) is a rare autosomal dominant systemic disease with an estimated prevalence of 1/6000. Renal angiomyolipoma (AML) is a benign tumour with high morbidity frequently present in TS. The aim of the study was to test the effect of rapamycin in reducing the volume of AML in TS.

**Methods:**

Twenty four-month prospective open-label, single arm, unicentre Phases II andIII study. The primary endpoint was to evaluate the effect of treatment on the reduction of at least 50% AML volume from baseline at 24 months. The secondary endpoints were: average tumour reduction, surgical complications, skin lesions and drug safety.

The study population comprised 17 patients, aged >10 years who were diagnosed with TS and had ≥1 renal AML >2 cm of diameter and had a serum creatinine < 2mg/dl and urine protein/creatinine ratio < 22.6 mg/mmol. The trial was conducted at Fundació Puigvert. Rapamycin was given to achieve stable plasma levels between 4 and 8 ng/ml. AML volume was estimated using orthogonal measurements by MRI at baseline, 6, 12 and 24 months.

**Results:**

Ten out of 17 patients were success responders for the main outcome −58.8%, 95%CI: 32.9% to 81.6%-. After 6 months of therapy, the mean volume decrease was 55.18% (5.01 standard error (SE); p<0.001) and 66.38% (4.41 SE; p<0.001) at year 1. There was no significant decrease between year 1 and 2. According to RECIST criteria, all patients achieved a partial response at year 1 and all but two had already achieved this partial response after 6 months.

The main analysis was performed according to the intention-to-treat principle analysis. Tumour volume was analyzed over time by means of mixed models for repeated measurement analysis. We used the baseline tumour volume as a covariate for the absolute change and percentage change from baseline data. The analysis was performed using SAS version 9.2 software, and the level of significance was established at 0.05 (two-sided).

**Conclusions:**

This study show that mTOR inhibitors are a relatively safe, efficacious and less aggressive alternative than currently available options in the management of AML in TS.

**Trial registration:**

EudraCT number: 2007-005978-30, ClinicalTrials.gov number: NCT0121712

## Background

Tuberous sclerosis (TS), or Bourneville-Pringle disease, is a rare autosomal dominant systemic disease with an estimated prevalence of 1/6000 [1].

Clinically, it manifests on the skin and may show renal, neurological, pulmonary and cardiac symptoms. The clinical presentation of TS ranges from few features of the disease in adults to severe neurological involvement in children [1].

Renal angiomyolipoma (AML) is a benign tumour formed by abnormal vessels, immature smooth muscle tissue, and adipocytes [2]. It is usually bilateral and multiple and its incidence ranges from 55% to 75% among TS patients. Morbidity is high, as it can lead to spontaneous haemorrhage and, albeit more rarely in very bulky AML, arterial hypertension and kidney failure [3]. This is the most serious manifestation after neurological involvement. The risk of rupture and spontaneous haemorrhage is generally related to the size of the AML and is particularly frequent when the tumour is greater than 3–4 cm [4]. In general, resection is avoided due to the loss of renal parenchyma and, therefore, kidney function. To date, the main therapeutic options are embolisation, elective surgery, and emergency nephrectomy in cases of uncontrollable haemorrhage [2].

TS is caused by mutations in genes *TSC1* and *TSC2*[5-7]. *TSC1* is responsible in a minimal percentage of cases, and causes the most benign forms of the disease [8]. It is located on chromosome 9q34, has 23 exons and codes for the protein hamartin. *TSC2* is located on chromosome 16p13, has 41 exons and codes for the protein tuberin. Tuberin and hamartin combine in an mTOR (mammalian target of rapamycin) regulatory pathway. mTOR is made up of two distinct complexes: mTORC1 and TORC2. The direct downstream targets of mTORC1; the eukaryotic initiation factor 4E-binding protein (4E-BP1) and ribosomal protein S6 kinase (S6K),tightly regulate the translational initiation machinery to control cell growth and proliferation. The mutations that lead to tuberin or hamartin absence or dysfunction give rise to disregulation of S6K and 4E-BP1 and to a loss of control of proliferation [9]. Rapamycin (Sirolimus, Rapamune®) is an immunosuppressive agent that inhibits mTOR, thus it is theoretically able to control cell growth and inadequate proliferation in patients with TS.

The objective of the present study was to demonstrate whether mTOR inhibitors, such as rapamycin, are a safe and effective therapeutic alternative to reducing the volume of AML in patients with TS.

## Material and methods

### Trial design

This trial was a 24-month, prospective, phase II-III study (EudraCT number: 2007-005978-30, ClinicalTrials.gov number: NCT0121712) conducted at Fundació Puigvert, Barcelona from July 2008 to May 2011. The trial was single-centre, non-controlled and non-blinded to test a new therapeutic indication in a marketed drug. It was carried out in accordance with the Declaration of Helsinki and the Good Clinical Practice guidelines of the International Conference on Harmonisation. The study was designed by the investigators and approved by the Fundació Puigvert Independent Ethics Committee and authorised by the Spanish Agency for Medicines and Medical Devices.

### Objectives

The primary objective was to evaluate the effect of rapamycin on the size of AML in patients with TS. The main outcome measure was the number of patients in whom a 50% reduction was observed in the size of the AML with the longest diameter compared to baseline.

The secondary objectives were the evaluation of the effect of treatment on volume reduction, the percentage of patients with surgical complications (bleeding, need for embolisation and/or surgery), skin lesions, and drug safety.

### Eligibility

The study population comprised 17 patients aged over 10 years who were diagnosed with TS and had at least 1 renal AML > 2 cm in diameter (irrespective of central nervous, cardiac, pulmonary, and/or cutaneous involvement) and baseline creatinine < 2 mg/dl. Written informed consent was provided in all cases. The Spanish TS patients association assisted with recruitment of patients and with study logistics such as patients’ travel and specimen shipping. Patients were recruited from all over Spain.

The exclusion criteria were: recent AML bleed, abnormal liver function or complete blood count, proteinuria (calculated as a protein-to-creatinine ratio > 22.6 mg/mmol), presence of active infection, history of surgery within 8 weeks before the start of treatment or neoplasm during the previous 2 years, fasting cholesterol > 7.8 mmol/l, low-density lipoprotein cholesterol > 5.1 mmol/l or triglycerides > 4.6 mmol/l, and a history of allergy to macrolides. All women underwent a pregnancy test before inclusion and at each follow-up visit.

### Follow-up and data collection

At the initial visit, patients received a dose of rapamycin (1 mg/d). Steady-state levels of rapamycin were determined by liquid chromatography-mass spectrometry in blood. Doses were increased by 1 milligram every 2 weeks until stable plasma levels (4–8 ng/ml) were achieved. Plasma samples were shipped every two weeks in order to determine rapamycin levels at Fundació Puigvert. These target rapamycin plasma levels were chosen based on the ones used for preventing rejection in renal transplant patients. Once reached, these levels were monitored at the follow-up visits (3, 6, 9, 12, 18 and 24 months of treatment). At each visit, patients underwent a physical examination, evaluation of adverse effects and adherence, and comprehensive blood workup (glucose, complete blood count, creatinine, MDRD, electrolytes, liver profile, bilirubin, lipids, and urinalysis with proteinuria calculated by ratio). Photographs of the skin lesions were taken. Abdominal MRI was performed at inclusion and at 6, 12 and 24 months.

In addition to the follow-up visits, each patient was also contacted on a monthly basis by telephone. Patients also had direct telephone access to the research team for any incidents that arose during the study period. Adverse events were recorded by evaluating the description, duration, and treatment of each incident, and the researcher determined whether there was a clear relation between the condition and the trial drug. Severe and unexpected adverse events were reported to healthcare authorities and the Independent Ethics Committee in accordance with the stipulations of Spanish Royal Decree 2004.

### Abdominal imaging evaluations

#### Image technique

Abdominal imaging evaluations were performed by 1.5 Tesla magnetic resonance (Vantage Atlas, Toshiba Medical Systems Corporation, Otawara-shi, Tochigi-ken, JAPAN) with a body phased-array coil. All studies were performed with the patient in supine position. Coronal, sagittal and axial scans were acquired with T1-weighted fast spoiled gradient echo and T2-weighted fast spin echo protocols with and without fat suppression.

#### Image analysis

Abdominal studies were analyzed by two independent radiologists with more than 10 years of experience interpreting abdominal imaging studies. Before the start of this study, the radiologists showed an intra and inter-observer variability of less than 5% in focal renal mass measurement. Tumour volume was estimated using orthogonal measurements, which assume that the masses are ellipsoid. In cases of AML with complex shapes, we used a standardised validated software program (Vitrea, Vital Imaging version 4.1.14.0.). When the tumour volume obtained by the two independent readers differed (always by less than 5%), a mean value of both determinations was calculated. In patients with multiple renal lesions, only the largest one was analyzed.

### Statistical methods

The sample size was set at 17 patients in order to have a statistical power of at least 80%. This would make it possible to detect a difference in the study group with respect to the efficacy expected in the untreated general population (0%) with a two-tailed 5% protection against alpha errors [10,11]. Ninety-five percent confidence intervals (95% CI) were calculated for the main binary outcome measure using exact methods, and missing data was attributed to failure. The results were discussed with regard to the previous population values observed for this disease. The main analysis was performed according to the intention-to-treat principle analysis including all patients (n=17). The analysis was also performed with the *per protocol* (PP) subset (including patients who complete the 24 moths period follow-up, n=13) to assess the robustness of the results.

Tumour volume was analyzed over time by means of mixed models for repeated measurement analysis (MMRM) [12], which assumes unmeasured observations to be missing at random (MAR) [13]. We used the baseline tumour volume as a covariate for the absolute change and percentage change from baseline analyzes. The analysis was performed using SAS version 9.2 software (SAS Institute Inc., Cary, NC), and the level of significance was established at 0.05 (two-sided).

The response was also post-hoc evaluated by the Response Evaluation Criteria in Solid Tumours (RECIST), i.e., considering success patients with a 30% tumour reduction [14].

### Limitations

As this study is non-controlled based on a small sample, it is affected by all the limitations associated with this methodology and with the interpretation of the results. This design was chosen due to the low prevalence of the disease and the limited availability of patients. The single-centre design was chosen in order to attempt to harmonise the criteria for the evaluation of response as much as possible.

## Results

### Patients and protocol completion

From July 2008 to May 2009, a total of 22 patients granted their consent to participate in the study. Of these, 17 were eligible for the study (8 men and 9 women) (Table 1). Three patients had undergone previous unilateral nephrectomy. The AML were bilateral in all patients and all patients also had a cranial MRI at the screening that showed no astrocytomas. No patient had been diagnosed with LAM (lymphangioleiomyomatosis). Eleven patients had a history of seizures and were on antiepileptic treatment. These patients needed higher doses of rapamycin to achieve the same plasma levels as the ones not taking antiepileptic drugs. The mean plasma rapamycin level was: 6.37 ng/ml (SD: 0.91 ng/ml, range: 4.77-8 ng/ml). For the subgroup of patients on antiepileptic drugs, this figure was 6.13 ng/ml (SD: 0.80 ng/ml, range: 4.8-7.7 ng/ml) and for those not taking antiepileptic drugs it was 6.8 ng/ml (SD: 0.99 ng/ml, range: 5.2-8 ng/ml). The mean dose given to patients was 4.9 mg per day (SD: 1.75 mg per day, range 2–7.75). For the subgroup of patients under antiepileptic drugs, the figure was 5.6 mg per day (SD: 1.80 mg per day, range: 3.25-7.75 mg per day) and for those not taking antiepileptic drugs it was 3.8 mg per day (SD: 1.09 mg per day, range: 2–4.75 mg per day). The means were calculated based on data from months 6, 12, 18 and 24.

**Table 1:** **Demographic and baseline data**^**a**^

	**N=17**
**Age (years), Mean (SD)**	31.8 (10.9)
**Sex (female)**	9 (52.9%)
**Brain**	
Cortical Tubers	14 (82.4%)
Seizures	11 (64.7%)
Cognitive Impairment	11 (64.7%)
Astrocytoma^b^	3 (17.7%)
**Skin**	
Facial angiofibroma	17 (100.0%)
Hipomelanotic macules	15 (88.2%)
Ungual fibroma	6 (35.3%)
Shagreen patch	9 (52.9%)
**Renal**	
Angiomyolipomas 5	1 ( 5.9%)
10	1 ( 5.9%)
Multiple	15 (88.2%)
Vascular embolization	3 (17.7%)
Nephrectomy	3 (17.64%)

^a^Descriptive statistics are frequencies and percentages or otherwise specified.

^b^Astrocytomas had been removed and were not present at the moment of trial initiation.

**Figure 1:**
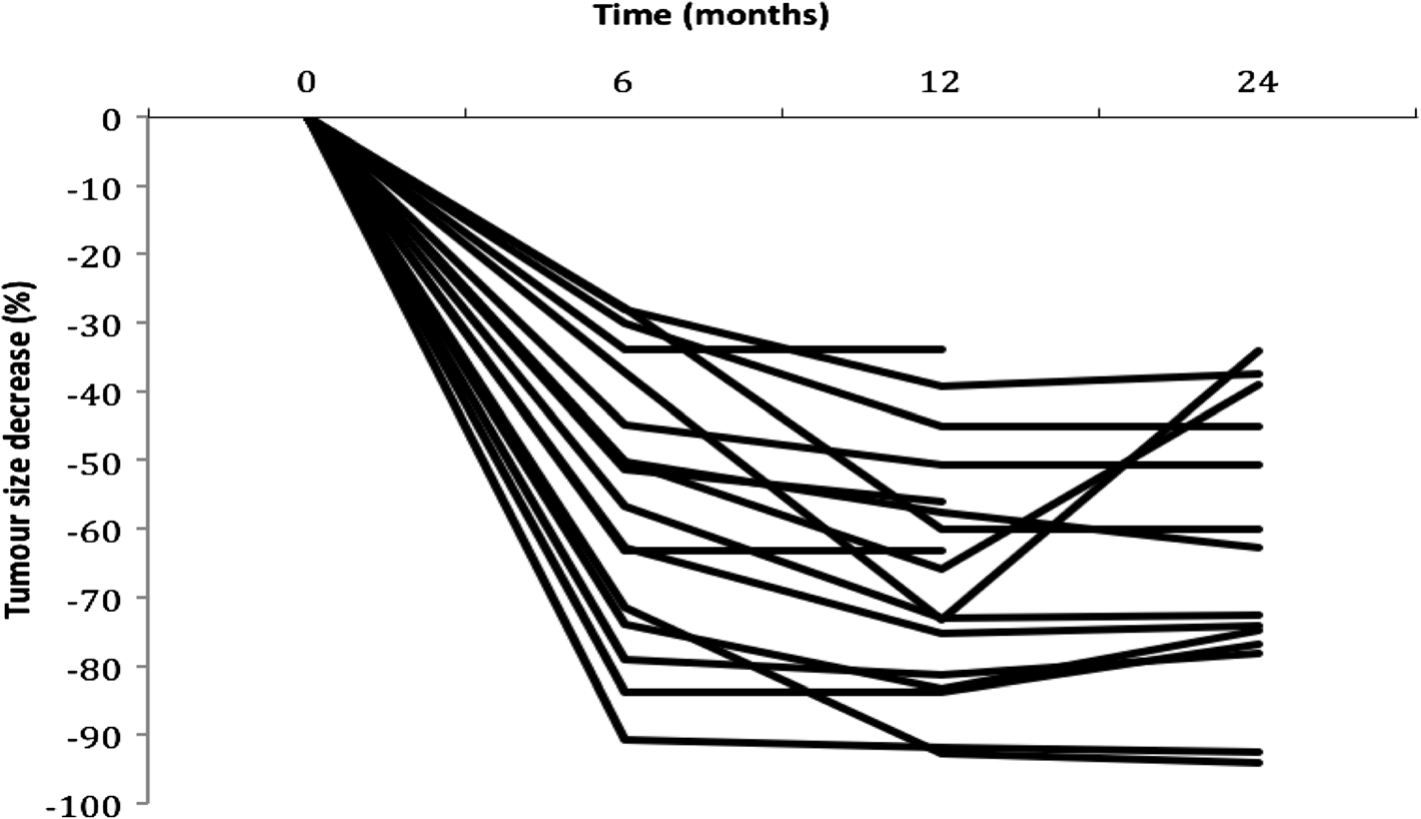
**Spaghetti plot for the individual percentage tumour decrease along time** .

Of the 17 patients included in the study, 16 completed the 12 months of treatment (Figure 1). Patient 4 was excluded at 10 months due to acute pyelonephritis and did not want to be rechallenged. Patient 17 was dechallenged at 14 months due to reactivation of the erythema nodosum that was present at inclusion and was not rechallenged, and patient 3 was withdrawn at 12 months of treatment due to nephrotic-range proteinuria that reverted after discontinuation of treatment. Patient 14 underwent major surgery due to infection of the frontal sinus and was dechallenged from month 13 to month 19.

### Outcomes

**Figure 2:**
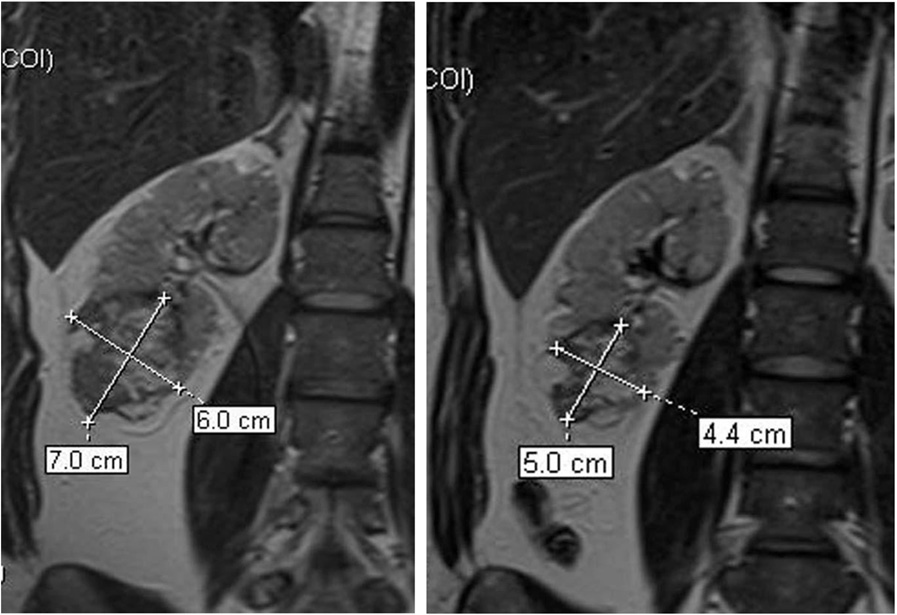
**Angiomyolipoma measured at baseline and after 6 months of treatment** .

Ten out of 17 patients achieved a 50% tumour reduction from baseline at 24 months (58.8%, [95%CI]: 32.92% to 81.56%) for the “intention to treat” (ITT) population, and 10 of 13 (76.9% [46.2%,95.0%]) for the PP subset (Table 2). The percentage decrease in the volume of the AML at 6, 12 and 24 months of treatment for each participant is shown in Figure 1. According to RECIST criteria, all patients achieved a partial response at one year and all but two had already achieved this partial response at 6 months (Figure 1, Table 2). Although the response persisted at 2 years the decrease in the percentage of volume was much more significant from time 0 to 1 year than from 1 to 2 years (see Table 2). In fact, there was no significant reduction in volume between 1 and 2 years, there were even some increases in volume. It is important to note that patient 14 had a MRI at 2 years but had not been taking rapamycin from month 13 to month 19 due to major surgery. In this patient, after 7 months of withdrawal and 4 months of rechallenge, the AML volume increased by 39.3% and remained similar to the volume at 6 months. Figure 2 shows a reduction in AML volume at 6 months .

**Table 2:** Efficacy results

**ITT population**	**(n=17)**	**Baseline**	**6 months**	**12 months**	**24 months**
**50% reduction from baseline**^1^	n (%) [95%CI]	-	11 (64.7%)	14 (82.4%)	10 (58.82%)
			[38.3% to 85.8%]	[56.6% to 96.2%]	[32.92% to 81.56%]
**30% reduction from baseline**^2^	n (%) [95%CI]	-	15 (88.2%)	17 (100.0%)	14 (82.4%)
			[63.6% to 98.5%]	[83.8% to 100.0%]	[56.6% to 96.2%]
**Adjusted baseline change (%)**	LSMean [95%CI]	0	−55.18 [−65.85 to −44.51]	−66.38 [−75.77 to −56.98]	−62.08 [−72.70 to −51.46]
	6 vs 12 months	-	Ref	−11.20 [−5.61 to −16.78] p<0.001	-
	12 vs 24 months	-	-	Ref	4.30 [−2.61 to 11.21] p=0.205
**Absolute values (cm3)**	LSMean [95%CI]	62.65 [24.05 to 101.25]	28.62 [12.91 to 44.33]	23.07 [8.29 to 37.84]	21.68 [11.30 to 32.06]
	0 vs 6 months	Ref	−34.03 [−9.90 to −58.16] p=0.008	-	-
	0 vs 12 months	Ref	-	−39.58 [−14.74 to −64.42] p=0.004	-
	0 vs 24 months	Ref	-	-	−40.97 [−11.15 to −70.79] p=0.010

Response of angiomyolipoma to rapamycin therapy: tumour volume and baseline reduction.

Figures for continuous variables are least square means (LSMean) and [95%CI] from the mixed models for repeated measurements (MMRM) analysis, for binary variables frequencies, (%) and [95%]. Values in the MMRM analysis are adjusted by the baseline volume, when specified.

^1:^ Main predefined outcome in the protocol. ^2:^ Reduction cut-off point according to the RECIST criteria.

No patient required AML surgery or embolization during the study.

**Figure 3:**
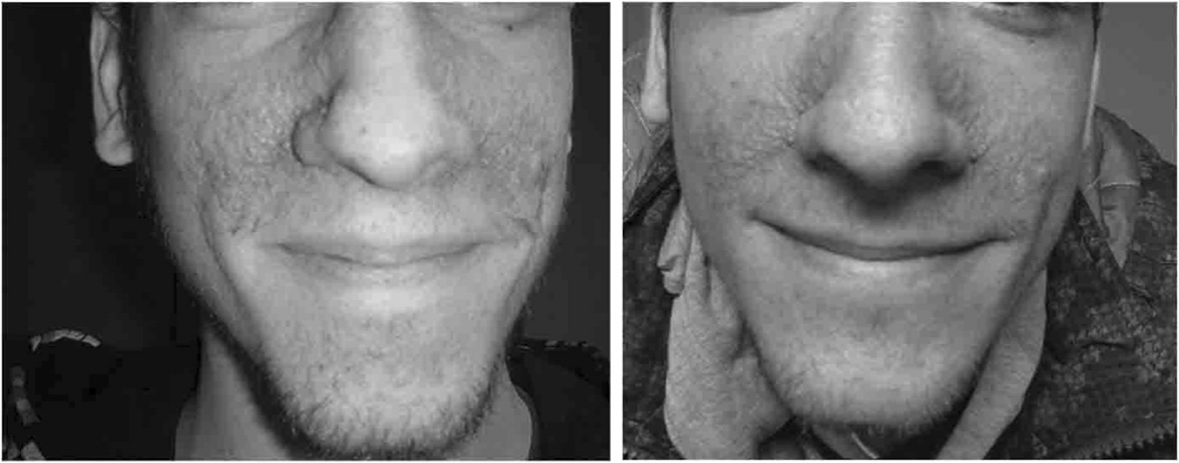
**Facial angiofibromas improvement after 2 years of therapy** .

Facial angiofibromas decreased in size and became paler and less rough (Figure 3). No objective quantification could be obtained. Although no changes in the shagreen patches or in the hypomelanic macules were detected, subjective improvement of periungual fibroma were reported. Although this was not a study endpoint, a slight improvement in the frequency of seizures was reported by some parents.

### Adverse events

The most common undesirable effects were as follows, and were considered mild: oral aphthous ulcers just after starting treatment (resolved with topical corticosteroids); hypertriglyceridaemia (controlled with medical treatment) (Table 3, Table 4); self-limiting episodes of diarrhoea; acneiform rash, microalbuminuria, microcytosis and hypochromia. Seven patients had increased their basal microalbuminuria and 9 did not show any signs of microalbuminuria (see Table 4). All these events were considered related to the treatment drug. Renal function remained stable during the study in all patients (Table 4). There were two severe adverse events that led to discontinuation of the treatment: nephrotic range proteinuria (appeared after 12 months of treatment, persisted with ACEI treatment and disappeared after rapamycin discontinuation) and exacerbation of erythema nodosum (appeared after 14 months of treatment and fully remitted after withdrawal). Both events were considered to be related to the treatment drug. One patient was hospitalised due to acute pyelonephritis, which was considered to be unrelated to the treatment. And another patient underwent major frontal sinus surgery which was also considered to be unrelated to the treatment drug.

**Table 3:** Total adverse events

	**No of patients (%) / No of events**
	**(N=17) / (N=36)**
**Gastrointestinal**	
Oral mucositis	5 (29.4%) / 7
Diarrhoea	3 (17.6%) / 5
**Skin-related**	
Acneiform rash	2 (11.8%) / 2
Exacerbation of erythema nodosum	1 ( 5.9%) / 2
nodosum	
**Metabolic or laboratory**	
Hypertriglyceridemia	5 (29.4%) / 5
Microcytosis and hypochromia	3 (17.6%) / 3
**Gynaecological**	
Menstruation disturbances	3 (17.6%) / 3
**Renal**	
Nephrotic range proteinuria	1 ( 5.9%) / 1
MAU <2 x ULN	3 (17.6%) / 3
MAU 2–3 x ULN	2 (11.8%) / 2
MAU 3 x ULN	2 (11.8%) / 2
Acute pyelonephritis	1 ( 5.9%) / 1

MAU: microalbuminuria.

ULN: upper limit of normality.

**Table 4:** Baseline and 24 months laboratory tests for renal function and lipids

**Patients**	**baseline creatinine**	**24 month creatinine**	**baseline proteinuria**^**1**^	**24 month proteinuria**^**1**^	**baseline cholesterol/**	**24 month cholesterol/**	**baseline triglycerides**	**24 month triglycerides**
	**(mg/dL)/MDRD (ml/min/1.73m2)**	**(mg/dL)/ MDRD (ml/min/1.73m2)**	**(mg/mmol)**	**(mg/mmol)**	**HDL/LDL**	**HDL/LDL**	**(mmol/L)**	**(mmol/L)**
					**(mmol/L)**	**(mmol/L)**		
1	0.84 /76	0.78/81	6.1	12.1	159/89/90	184/74/101	45	52
2	0.93/>90	0.95/>90	22.4	40.0 ^5^	118/46/72	167/54/100	43	63
3	1.22/74		22.5	^**2**^	205/74/131		86	
4	0.96/71		10.3	^**3**^	176/83/94	^**3**^	144	^**3**^
5	0.99/87	0.97/88	9.1	8.2	202/63/139	181/44/125 ^4^	116	64
6	0.67/>90	0.58/>90	5.0	9.6	240/86/156	152/63/89 ^4^	198	116
7	1.15/50 ^0^	0.98/60	9.4	28.6 ^5^	192/78/114	188/77/102	47	47
8	1.07/83	1.09/80	5.6	4.0	154/56/98	202/66/120	62	77
9	0.77/>90	0.86/>90	13.2	47.0 ^5^	125/36/90	226/52/136 ^4^	102	186
10	0.85/78	0.77/87	5.0	4.3	212/55/157	167/75/82 ^4^	48	51
11	0.71/>90 ^0^	0.86/78	7.7	9.4	94/40/54	118/41/64	76	62
12	0.42/> 90	0.48/>90	13.3	11.1	142/38/104	193/42/127	117	120
13	0.62/>90	0.61/>90	6.6	9.8	183/96/87	163/84/90	53	41
14	0.83/>90	1.01/>90	6.4	8.9	203/83/120	176/39/104 ^4^	175	163
15	0.68/>90	0.69/>90	9.0	18.2	200/78/122	170/75/80	66	73
16	0.62/>90	0.54/>90	12.9	11.9	156/51/105	120/49/50 ^4^	145	104
17	1.30/42 ^0^	1.24/44	22.4	32.3 ^5^	292/106/189	216/69/147 ^4^	243	117

0-Patients 7, 11, 17 had undergone a nephrectomy at least one year before the start of the trial.

1-Expressed as a protein-to-creatinine ratio.

2-Patient 3 was withdrawn at 12 months of treatment due to nephrotic-range proteinuria that reverted after discontinuation of treatment.

3-Patient 4 was excluded at 10 months due to acute pyelonephritis and did not want to be rechallenged.

4-Statins were prescribed in patients 5, 6, 9, 10, 14, 16, 17.

5- ACEI were prescribed for microalbuminuria in patients 2, 7, 9, 17.

## Discussion

This study demonstrates that mTOR inhibition through the use of rapamycin is effective in reducing AML volume in TS patients and has an acceptable safety profile.

The drug is marketed and indicated for prophylaxis of acute rejection in adult kidney transplant recipients with low immunological risk.

Preliminary clinical data suggest that rapamycin could play a beneficial role in the treatment of TS [15,16]. There are case reports in which the size of astrocytomas or angiomyolipomas decreased in patients with TS and good tolerability was reported [17-21]. Also some clinical trials showed beneficial effect of mTOR inhibitors in lymphangioleiomyomatosis [22] and astrocytomas [23]. Moreover, 3 clinical trials show evidence of AML response to mTOR inhibition [24-26]. In a non-controlled, phase II trial, Bissler JJ et al. showed a reduction in the size of renal angiomyolipomas (53.2% ± 26.6%) in 14 TSC patients with AML larger than 1 centimetre in size treated over 1 year [24] . Davies DM et al. also carried out a non-controlled trial with 16 patients with AML and TSC or LAM. Seven patients with TSC and AML reached 2 years of treatment with sirolimus and the results showed a significant reduction of AML burden [25]. The response rate, by RECIST criteria, was 50%, being all partial responses Very recently Dabora SL et al. reported their experience in treating 36 patients with TSC or LAM with Sirolimus for one year and had a 44.4% response rate (partial responses in all cases) according to RECIST criteria [26]. We also reported the preliminary results at one year observing an excellent response to treatment [27]. An explanation for the higher success rates in our trial compared to others may be that we focused the evaluation of AML volume decrease in the largest AML while others analyzed the AML in general. Perhaps, the greater AML have a better response to treatment than smaller ones, based on its hypothetically higher mitotic rate.

The hypothesis of this trial is that inhibitors of the Akt signalling cascade, such as rapamycin, can block the inadequate proliferation observed in TS in the form of AML [28]. The drug reduces vascular endothelial growth factor (VEGF) plasma levels and, taking into account the considerable tumour vascularisation in TS caused by up-regulation of VEGF, its anti-angiogenic action could prove very beneficial [29].

This study is the largest and longest carried out to date focused on AML in patients with TSC without LAM. The results show that AML shrank faster early on in the trial and persist with approximately the same volume while on treatment. There is a significant decrease in volume after six months of treatment, which continues at a slower rate up to one year, but remains stable from 1 to 2 years (RECIST partial response rate: 100% at one year). This evidence suggests that the shrinkage power of the drug is at its maximum at the beginning, probably due to its anti-angiogenic effect. And, although there does not seem to be any beneficial effect after one year, there is indeed, as some authors demonstrated by withdrawing the treatment and then showing an increase in AML volume [24,26]. Therefore, it seems reasonable to assume that TSC patients with large AML should be kept under mTOR inhibition for life. This suggestion may raise two concerns: what the optimal dose is and whether we can accept the side effects of the drug for a lifetime. In this study, we experienced a great deal of trouble achieving the desired target plasma levels of sirolimus in patients on antiepileptic treatment, which is very common among TSC patients. Moreover, all patients in general needed higher doses than transplanted patients to achieve the same target plasma levels. Larger studies and a long follow-up period should address whether low doses of mTOR inhibitors are enough to prevent AML growth after the initial reduction. The most frequent adverse reactions we recorded were stomatitis (27.7%), which was observed at the start of treatment. The reaction was dose-dependent and easily managed with topical corticosteroids and dose adjustment. The next most frequent reaction was hypertriglyceridaemia (22.2%) mainly in patients who already had high triglyceride levels prior to inclusion. Medical treatment was provided in most cases. Given the anti-proliferative effect of rapamycin, we also observed microcytosis and hypochromia (16.6%), with normal results for iron metabolism and stable haemoglobin values. A total of 41% of patients developed microalbuminuria but no patients developed impaired renal function. However, the development of microalbuminuria raises the concern on the long term effects on renal function of mTOR inhibition. Two patients were withdrawn because of serious adverse events, one due to reactivation of erythema nodosum and the other due to nephrotic-range proteinuria, which fully resolved after discontinuation of the treatment.

There are efficacy reports on topical and oral rapamycin for facial angiofibromas in TSC patients [30,31], however this clinical feature has not been analyzed in previous trials using systemic rapamycin. In this study, we noticed an improvement in facial angiofibromas and a subjective improvement of periungual fibromas.

## Conclusions

The results of this study show that mTOR inhibitors are a relatively safe and effective therapeutic alternative in the management of AML in patients with TS. These agents represent a therapeutic alternative that is less aggressive than the options currently available and, above all, compared with the available existing therapies, is expected to preserve renal function.

The efficacy and acceptable safety profile of mTOR inhibitors in patients with TS shown in this trial and the other 3 trials mentioned above make mTOR inhibitors a drug with high potential for becoming a first-line treatment in TSC. However, individualised treatment is expected; mTOR inhibitors are not harmless and customised therapy is anticipated in those patients with AML with high chances of bleeding.

## Competing interests

The authors had no involvements that might raise the question of bias in the work reported or in the conclusions, implications, or opinions stated.

## Authors’ contributions

CC did most of the patient’s follow up and participated in the whole study. TM and VC did the MRI interpretations. FT did the statistical analysis. RT, SM, JB designed the trial and followed it up. All authors contributed to elaborating the manuscript. All authors read and approved the final manuscript.

## Author’s information

RT chairs the Inherited Renal Diseases Unit at Fundació Puigvert. She also chairs the working group on inherited kidney disorders within the Spanish Society of Nephrology and is the President of the Scientific Committee for the AIRG-E.
